# Reduced CAG Repeats in the Androgen Receptor Gene May Independently Cause Polycystic Ovarian Syndrome

**DOI:** 10.3390/cimb48050526

**Published:** 2026-05-18

**Authors:** Rhea Sharma, Daniel H. Shain

**Affiliations:** Biology Department, Rutgers The State University of New Jersey, Camden, NJ 08103, USA; rrs167@scarletmail.rutgers.edu

**Keywords:** polycystic ovarian syndrome, androgen receptor, CAG repeat polymorphism, hyperandrogenism, gonadotropin regulation

## Abstract

Polycystic ovarian syndrome (PCOS) affects over 116 million women globally and is typically linked with excess androgens such as testosterone. Many patients, however, display classic PCOS symptoms despite normal serum androgen. One proposed mechanism for these cases involves a shortened CAG (i.e., encodes glutamine) repeat length in the androgen receptor (AR) gene, which increases AR activity without elevating testosterone. Fewer glutamine repeats alter the AR’s N-terminal domain and may contribute to strengthened interactions with co-activators and enhanced transcription of androgen-regulated genes. Heightened AR activity in hypothalamus neurons stimulates increased pulsatile release of gonadotropin-releasing hormone (GnRH), which disrupts pituitary secretion dynamics and favors luteinizing hormone (LH) over follicle-stimulating hormone (FSH). This altered LH/FSH ratio leads to impaired folliculogenesis, anovulation and other hallmark PCOS symptoms. Targeting AR activity directly, for example by using compounds that covalently modify the AR N-terminal domain to suppress activity, may therefore offer a more precise treatment strategy for PCOS.

## 1. Introduction

Between 10 and 13% of reproductive-aged women worldwide are affected by polycystic ovarian syndrome (PCOS), and up to 70% of women affected are undiagnosed [1]. PCOS is characterized by varying combinations of ovulatory dysfunction, hyperandrogenism, and polycystic ovarian morphology [2]. Women may also experience insulin resistance and infertility. According to the 2003 Rotterdam criteria, diagnosis of PCOS must meet at least two of the following criteria: oligo-anovulation, hyperandrogenism or polycystic ovaries [2]. Oligomenorrhea is typically defined as menstrual cycles longer than 35 days or fewer than 8–9 menses per year, depending on the guideline. Ovulatory dysfunction may also include amenorrhea or biochemical evidence of anovulation. Hyperandrogenism can be diagnosed clinically by hirsutism, acne and/or alopecia, or biochemically by measuring serum testosterone levels. Polycystic ovaries can be diagnosed by ultrasonography.

The Rotterdam criteria classify PCOS into four phenotypes. Phenotype A is characterized by hyperandrogenism, ovulatory dysfunction and polycystic ovarian morphology and is associated with the highest metabolic risk, including insulin resistance, dyslipidemia and metabolic syndrome [3]. Phenotype B includes hyperandrogenism and ovulatory dysfunction but lacks polycystic ovarian morphology [3]. Phenotype C is defined by hyperandrogenism and polycystic ovarian morphology, while Phenotype D includes ovulatory dysfunction and polycystic ovarian morphology without hyperandrogenism [3].

Almost two-thirds of PCOS cases involve hyperandrogenism [3]. For this reason, PCOS treatments such as the oral contraceptive pill may be administered to reduce androgen levels [4]. But at least 20–40% of patients who present PCOS symptoms also have normal circulating androgen levels. The mechanism by which PCOS develops in patients with normal androgen levels remains unclear. We propose here that hyperandrogenic symptoms may independently arise from increased androgen receptor (AR) activity, as a consequence of variable numbers of CAG repeats in the AR gene.

## 2. Role of Androgens in PCOS

**Figure 1:**
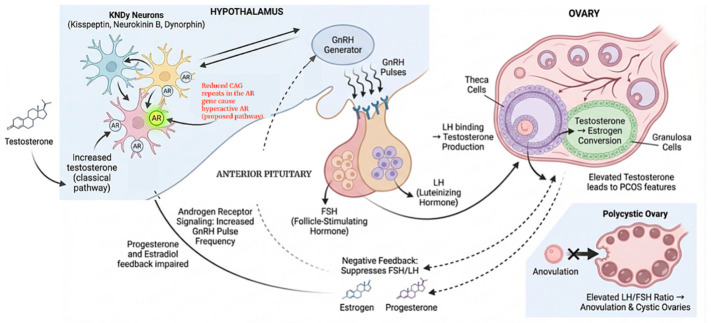
Neuroendocrine regulation of ovulation and polycystic ovarian morphology. KNDy neurons modulate hypothalamic GNRH pulsatility, influencing anterior pituitary secretion of FSH and LH. LH stimulates testosterone production, which is converted to estrogen by granulosa cells. Elevated testosterone disrupts the normal feedback from progesterone and estrogen, increasing GnRH pulse frequency and LH/FSH ratio, leading to anovulation and polycystic ovaries (classical pathway). However, overactive androgen receptors (ARs) due to shorter CAG repeats can lead to elevated LH/FSH ratios even when testosterone levels are normal (proposed pathway).

The arcuate nucleus, a region in the hypothalamus, contains subpopulations of kisspeptin, neurokinin B and dynorphin (KNDy) neurons. These project directly to the hypothalamic gonadotropin-releasing hormone (GnRH) generator, which releases GnRH in pulses or bouts. GnRH then binds to receptors in the anterior pituitary gland, stimulating the release of follicle-stimulating hormone (FSH) and luteinizing hormone (LH). LH binds to theca cells in the ovaries, triggering the release of testosterone, which binds to ovarian granulosa cells and is converted into estrogen via FSH aromatase stimulation [5]. Estrogen and progesterone regulate gonadotropin secretion by negative feedback loops at both the hypothalamus and the pituitary. In PCOS, impaired progesterone and estradiol negative feedback is thought to contribute to persistently rapid GnRH pulsatility [6]. Androgens modulate hypothalamic neuronal networks involved in GnRH pulse generation, including KNDy-related circuits, thereby contributing to altered reproductive neuroendocrine signaling [7]. Elevated testosterone levels are associated with more frequent release of GnRH in pulses and also reduce hypothalamic sensitivity to negative feedback from progesterone and estradiol [8]. In response to increased GnRH pulsatility, the pituitary gland preferentially releases LH over FSH, resulting in elevated serum LH/FSH ratios in most PCOS patients [8] (Figure 1).

An ovarian follicle comprises granulosa and theca cells surrounding a central oocyte. The growth of follicles occurs in different stages, influenced by factors secreted by the oocyte and androgen concentration. For instance, oocyte-derived growth factors such as GDF9 and BMP15 regulate granulosa cell growth, differentiation and follicular development through paracrine signaling [9]. Androgens may support early follicular growth and increase granulosa cell responsiveness to FSH; however, excess androgen in PCOS is associated with disordered folliculogenesis and follicular arrest [10]. Normally, FSH binds to granulosa cells, increasing the concentration of cyclic AMP (cAMP) and promoting differentiation [11]. As the follicle develops, the oocyte matures, eventually being released during ovulation. In cases of hyperandrogenism, however, reduced FSH levels contribute to arrested follicular development, and thus many women with PCOS have small antral follicles and experience anovulation [12].

Anti-Müllerian hormone (AMH) also plays a role in follicular development. Evidence suggests that AMH increases GnRH pulse frequency, causing elevated LH and testosterone production [13]. Women with PCOS often have serum AMH levels approximately two- to threefold higher than controls [14], likely reflecting an increased number of preantral and small antral follicles as well as altered granulosa cell function. Elevated androgen may also keep AMH levels above normal.

Elevated testosterone levels are frequently associated with obesity. Approximately 38% of women with PCOS are overweight or obese [15]. In fact, insulin resistance affects an estimated 57–95% of women with PCOS [16]. Obesity contributes to hyperinsulinemia, which activates cytochrome P450c17α, a key enzyme associated with androgen biosynthesis, thereby increasing testosterone production [15]. Hyperinsulinemia also enhances LH–mediated stimulation of theca cells and reduces hepatic production of sex hormone-binding globulin (SHBG), resulting in an increased bioavailability of free testosterone [17].

PCOS phenotypes A–C are commonly associated with hyperinsulinemia and metabolic syndrome, whereas phenotype D exhibits lower levels of insulin resistance and dyslipidemia [18]. However, women with phenotype D who are obese demonstrate metabolic features similar to other phenotypes, suggesting that obesity-related insulin resistance plays a central role in the development of these metabolic abnormalities [19].

PCOS may also be linked to prenatal testosterone exposure during the 18th week of gestation, acting through AMH. Women with PCOS display elevated AMH levels [20], and mice models show that excess AMH increases maternal testosterone [21]. Consequently, female offspring have more active GnRH neurons and a PCOS-like phenotype, which persists across generations [21]. Thus, epigenetic modifications during fetal development may continue into adulthood and influence the onset of PCOS. Indeed, maternal testosterone levels correlate positively with AMH in both PCOS adolescents and adults [13].

Puttabyatappa and Padmanabhan (2018) propose a two-hit model in which PCOS originates in utero through exposure to excess androgens and AMH, which program long-term neuroendocrine dysfunction [22]. The prenatal “first hit” is amplified by a “second hit” of adverse maternal metabolic factors, including obesity, hyperglycemia and hypertension. Likewise, endocrine-disrupting chemicals such as bisphenol A (BPA), commonly found in plastics, are associated with increased androgen synthesis in theca cells [23], leading to hyperactivity of the GnRH pulse generator in rat models [24]. Note that women with PCOS have significantly higher BPA levels compared with controls and that the association between BPA and testosterone levels is higher in obese women [25].

## 3. Androgen Receptor and Glutamine Repeats

Testosterone binds to androgen receptors (ARs) found in the hypothalamus and granulosa cells in the ovaries. Cytoplasmic ARs dimerize upon association with androgens and translocate into the nucleus where they bind to androgen response elements (AREs). Many AREs function as distal enhancers and promoters.

**Figure 2:**
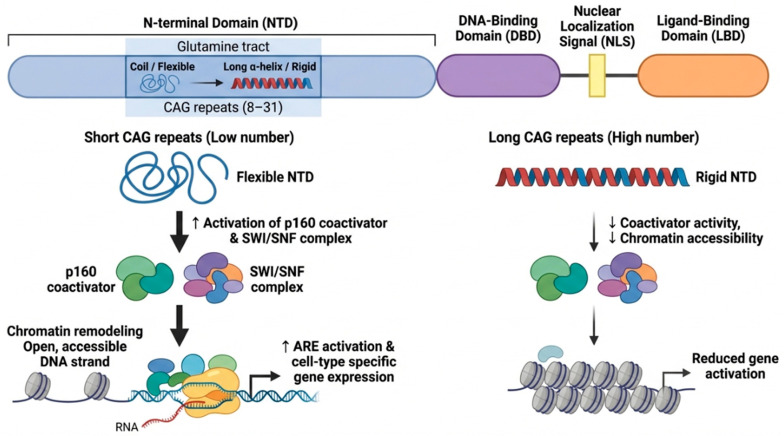
Impact of CAG repeat length in the androgen receptor (AR) N-terminal domain. The N-terminal domain contains a polymorphic glutamine tract encoded by CAG repeats. A lesser number of CAG repeats increases chromatin accessibility and enhances AR interactions with co-regulators and co-activators, resulting in higher gene expression, clinically leading to hyperandrogenic symptoms such as hirsutism and/or anovulation.

The AR protein contains an N-terminal domain, a DNA-binding domain, a nuclear localization signal, and a C-terminal ligand-binding domain (LBD) [26]. A variable number of CAG repeats between 8 and 31 encode a glutamine tract in the N-terminal region [27,28]. Crystal structures of the N-terminal are not determined, but circular dichroism and nuclear magnetic resonance show that CAG repeats lead to increased α-helical structure [29]. Since the AR interacts with nuclear transcription factors and co-regulators, the number of CAG repeats may influence the formation of the transcription regulatory complex (Figure 2). For example, in vitro studies show that shorter glutamine tracts lead to increased activities of the p160 coactivator and SWI/SNF remodeling complex [30]. Both p160 and SWI/SNF facilitate transcription factor assembly and remodel chromatin, making gene targets more accessible to RNA polymerase [31]. In contrast, increased CAG repeats suppress AR activity by causing AR aggregation, thus preventing formation of the transcription initiation complex [32]. Low numbers of CAG repeats appear to increase flexibility of the N-terminal domain, thereby enabling AR to activate AREs and cell type-specific gene expression [33]. Note that long GGN repeats (encoding polyglycine) in the N-terminal domain of AR have also been associated with reduced transcriptional activity [34], but this mechanism remains mostly unexplored.

The role of expanded CAG/glutamine repeats in human disease is not unique. Huntington’s disease (HD) is characterized by 36 or more CAG repeats that form RNA hairpin structures resistant to degradation and that interact abnormally with other proteins [35]. Abnormal protein sequestration is also observed in spinocerebellar ataxias (SCA), a neurodegenerative disorder characterized by CAG expansion in multiple genes [36]. Non-coding RNAs with CAG repeats sequester RNA-binding proteins responsible for translocating mRNA transcripts [36]. Spinal and bulbar muscular atrophy (SBMA) is a motor disease caused by CAG length expansion in exon 1 of the AR gene. Expanded polyglutamine tracts in the AR N-terminal domain can aggregate with cytochrome c oxidase subunit Vb (COXVb), which functions in the electron transport chain, leading to mitochondrial dysfunction [37]. ARs with exceptionally long glutamine tracts sequester cAMP response element-binding protein (CREB), which disrupts RNA polymerase II-dependent transcription [38]. Thus, increasing CAG repeat number in various genes often has negative effects.

CAG repeat length among SBMA patients tends to become longer between parent and child [39]. Interestingly, the age of SBMA onset inversely correlates with CAG length [39]. CAG length also expands across somatic and germline cells for patients with HD [40]. In contrast with highly unstable repeat expansion disorders, AR CAG repeat length in PCOS appears to be relatively stable across generations [41].

## 4. Current Data and Limitations on CAG Repeat Length Analysis

Some analyses find no significant differences between the length of CAG repeats and PCOS [42], while others find that PCOS patients with elevated androgen levels have lower CAG repeat numbers [42,43,44]. Several limitations should be considered when interpreting the current literature. Many studies are constrained by relatively small sample sizes and geography, as participants are often recruited from a single region. Additionally, PCOS diagnoses are based on different criteria (e.g., ultrasound morphology, biochemical evidence of hyperandrogenism), and comparability across studies is limited by a lack of phenotypic stratification. In many cases, patient groups are not distinguished by androgen status (hyperandrogenic vs. normoandrogenic) or by specific PCOS phenotypes. Thus, analyses include heterogeneous populations, which can obscure meaningful associations and contribute to conflicting data. Currently, no direct clinical test distinguishes AR-hypersensitivity-mediated PCOS from classic hyperandrogenic PCOS in normoandrogenic patients. Prospective studies measuring LH pulse frequency and AR transcriptional activity in genotype-stratified patients will be necessary to validate this mechanism.

## 5. CAG Repeats and Normoandrogenic Development

Examining PCOS in the context of testosterone levels may provide additional clues about inconsistencies in clinical data. For example, X chromosomes containing shorter numbers of CAG repeats in PCOS patients were preferentially active (i.e., not inactivated; [43]. Likewise, expression of the longer CAG length allele increased concordantly with serum testosterone levels [45]. Taken together, this is consistent with normal to low serum testosterone level PCOS patients being more likely to express AR gene variants with fewer numbers of CAG repeats. Phenotypically, shorter CAG repeat length also correlates with more severe androgenic features such as increased hirsutism scores, acne and oligomenorrhea [45,46].

## 6. Implications for Treatment

Combined oral contraceptives (COCs) are a first-line therapy for PCOS due to their ability to suppress androgen production and regulate menstrual cycles. COCs suppress luteinizing hormone (LH) secretion via a negative feedback loop at the hypothalamic–pituitary axis, thereby reducing ovarian androgen synthesis [47]. Likewise, oral contraceptives containing ethinylestradiol increase hepatic production of sex hormone-binding globulin (SHBG), which binds circulating testosterone and reduces free androgen bioavailability [48].

COCs provide exogenous estrogen and progestin in a predictable, cyclic manner, compensating for the hormonal irregularities associated with oligo-ovulation and anovulation. Chronic anovulation and reduced progesterone exposure increase the risk of endometrial hyperplasia and malignancy in women with PCOS [15]. The progestin component mimics endogenous progesterone, stabilizing the endometrium and inducing regular withdrawal bleeding, thereby reducing the risk of endometrial cancer associated with prolonged unopposed estrogen exposure [15].

Metformin is frequently prescribed alongside oral contraceptives to address metabolic dysfunction and insulin resistance associated with PCOS. Metformin inhibits mitochondrial complex I, leading to reduced ATP availability and suppression of hepatic gluconeogenesis, which enhances glucose clearance and improves insulin sensitivity [49]. As a result, metformin lowers fasting glucose and insulin levels, contributing to improved metabolic control [50,51]. Beyond its metabolic effects, metformin also reduces circulating androgen levels through both direct and indirect mechanisms. Indirectly, decreased hyperinsulinemia increases hepatic SHBG production, further reducing free androgen concentrations [51]. Lower insulin levels also diminish LH-stimulated androgen production by ovarian theca cells [52]. Directly, metformin suppresses ovarian steroidogenesis by reducing the expression and activity of 3β-hydroxysteroid dehydrogenase (HSD3B2) and 17α-hydroxylase/17,20-lyase (CYP17A1), key enzymes responsible for converting steroid precursors into androgens [53].

PCOS patients with phenotype D are normoandrogenic and associated with less severe metabolic syndrome. Limited evidence on the efficacy of oral contraceptives and metformin is available on this subset of patients. Reducing androgen, however, may not be effective in women with an overactive AR protein or in cases with shortened glutamine repeats, since this mechanism functions separately from androgen-induced PCOS.

An effective approach for treating androgen-independent PCOS may be anti-androgens such as Spironolactone and Flutamide, which both work as AR antagonists [54]. Spironolactone is a competitive AR antagonist and 5a-reductase inhibitor [55]. Flutamide also competitively inhibits testosterone binding to AR but is not recommended due to liver toxicity [55]. Cyproterone acetate also acts as an AR antagonist but carries the risk of meningioma [55].

## 7. Conclusions and Future Directions

Between 20 and 40% of PCOS patients have normal androgen levels. Among androgen-independent PCOS cases, shorter tracts of the N-terminal glutamine repeats may amplify AR activity despite normal androgen levels. In vitro studies suggest that shorter CAG repeat length is associated with increased AR activity; however, clinical evidence remains conflicted regarding whether patients with PCOS consistently exhibit shorter CAG repeats. Stratifying patients based on androgen status—particularly distinguishing between hyperandrogenic and normoandrogenic PCOS (e.g., Phenotype D)—may help clarify these inconsistencies. Future research should also confirm if circulating testosterone levels affect X-chromosome inactivation patterns of the AR gene in PCOS patients. If so, it will be important to determine whether standard therapies, including combined oral contraceptives and metformin, are equally effective in PCOS patients with shorter CAG repeat lengths or whether AR CAG genotyping could inform more personalized, genotype-guided treatment strategies. Finally, therapeutic approaches that directly target the AR pathway should be pursued along with ways to assess their efficacy within relevant experimental and clinical models.

## Data Availability

No new data were created or analyzed in this study. Data sharing is not applicable to this article.

## Abbreviations

The following abbreviations are used in this manuscript:

AREs	androgen response elements
AMH	anti-Mullerian hormone
AR	androgen receptor
BPA	bisphenol A
cAMP	cyclic AMP
COCs	combined oral contraceptives
COXVb	cytochrome c oxidase subunit Vb
CREB	cAMP response element-binding protein
FSH	follicle stimulating hormone
GNRh	gonadotropin-releasing hormone
HD	Huntington’s Disease
KNDy	kisspeptin, neurokinin B, and dynorphin
LBD	ligand-binding domain
LH	luteinizing hormone
PCOS	polycystic ovarian syndrome
SCA	spinocerebellar ataxias
SHBG	sex hormone-binding globulin
SBMA	spinal and bulbar muscular atrophy
